# Ectopic Kidney With Vesicoureteral Reflux and Associated Gonadal and Müllerian Anomalies in a Prepubertal Child: A Case Report

**DOI:** 10.7759/cureus.106884

**Published:** 2026-04-12

**Authors:** Sandeep Sen, Uma K Dutt, Sunil Thakur, Bijaya Kumar Sethi

**Affiliations:** 1 Pediatric Surgery, All India Institute of Medical Sciences Bilaspur, Bilaspur, IND; 2 Urology, All India Institute of Medical Sciences Bilaspur, Bilaspur, IND; 3 Anesthesiology, Dr. Radhakrishnan Government Medical College, Hamirpur, IND

**Keywords:** ectopic kidney, laparoscopy, mullerian anomaly, unicornuate uterus, vesicoureteric reflux

## Abstract

The association between congenital renal abnormalities and Müllerian duct anomalies (MDAs) has long been recognized. We present an unusual case, which is of a female child with left grade 4 vesicoureteric reflux (VUR) with ectopic pelvic kidney, who underwent laparoscopic extravesical ureteric reimplantation. Incidental findings on laparoscopy were a left-sided unicornuate uterus placed below the iliac vessels and an ectopic right ovary placed near the deep inguinal ring, which was missed on imaging. Parents of the patient were counselled regarding the complications associated with Müllerian anomalies, like menstrual irregularities, infertility, ectopic pregnancy, and the need for regular follow-up in the future.

## Introduction

Müllerian duct anomalies (MDAs) are deviations from the normal anatomy involving the uterus, fallopian tubes, and/or vagina, which are derived embryologically from the Müllerian ducts [1]. Forty percent of patients have coexisting anomalies of renal, ureter, and bladder due to the interlinked embryology of paramesonephric ducts, mesonephric ducts, and urogenital sinus [2]. Renal agenesis is one of the most common anomalies, which is associated with MDAs with a reported incidence of 30% [3], and other associated abnormalities include malrotation, pelvic kidneys, and dysplastic kidneys [3]. A strong association of renal ectopia with reproductive structure anomalies is found in 15-45% patients [4]. Bicornuate or unicornuate uterus with atresia of one horn, rudimentary or absent uterus, proximal and/or distal vagina, and duplication of vagina are found in 20-66% of female patients [3]. Here, we present the case of an 11-year-old female with a right ectopic pelvic kidney with left grade-4 vesicoureteric reflux (VUR), with an incidentally diagnosed unicornuate uterus and ectopic right ovary while performing laparoscopic extravesical ureteric reimplantation.

## Case presentation

**Figure 1:**
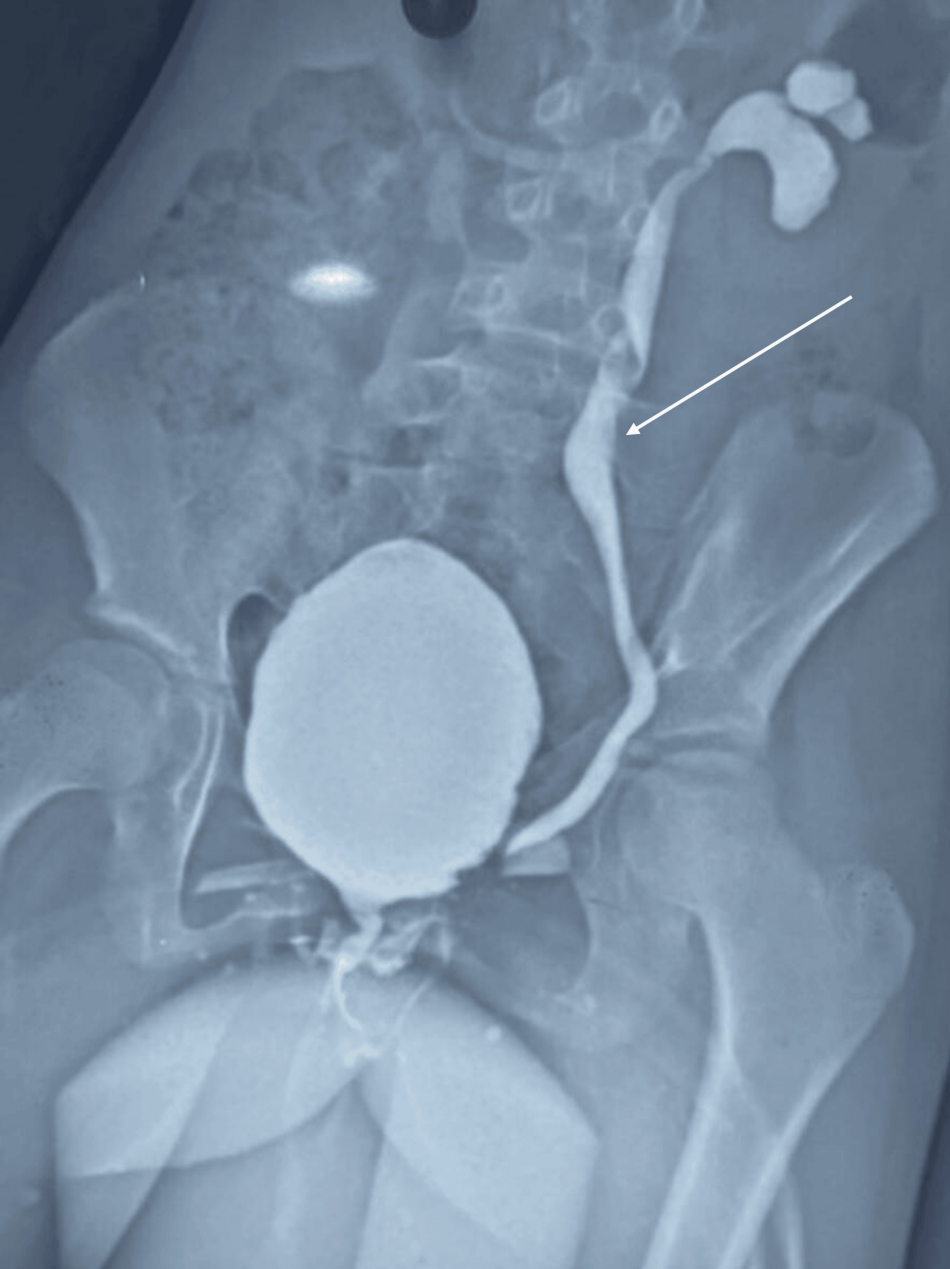
Preoperative MCUG showing grade 4 VUR The white arrow points to grade 4 VUR. MCUG, micturating cystourethrogram; VUR, vesicoureteric reflux.

An 11-year-old female child presented to the OPD with chief complaints of pain in the left lumbar region, fever, and burning micturition. The patient was prepubertal with a sexual maturity rating of 1, corresponding to Tanner stage 1. The patient was admitted and evaluated for a urinary tract infection (UTI). Urine microscopy showed numerous pus cells, and urine culture sensitivity showed growth of *Escherichia coli*. Ultrasound (USG) abdomen showed right pelvic ectopic and left orthotopic kidney with no hydronephrosis in any of the kidneys, with echoes in the bladder. UTI was managed with intravenous antibiotics as per sensitivity. The patient had a history of similar episodes in the past, which were managed in a local public hospital. A micturating cystourethrogram (MCUG) was done, which showed features suggestive of left grade-4 VUR (Figure 1).

**Figure 2:**
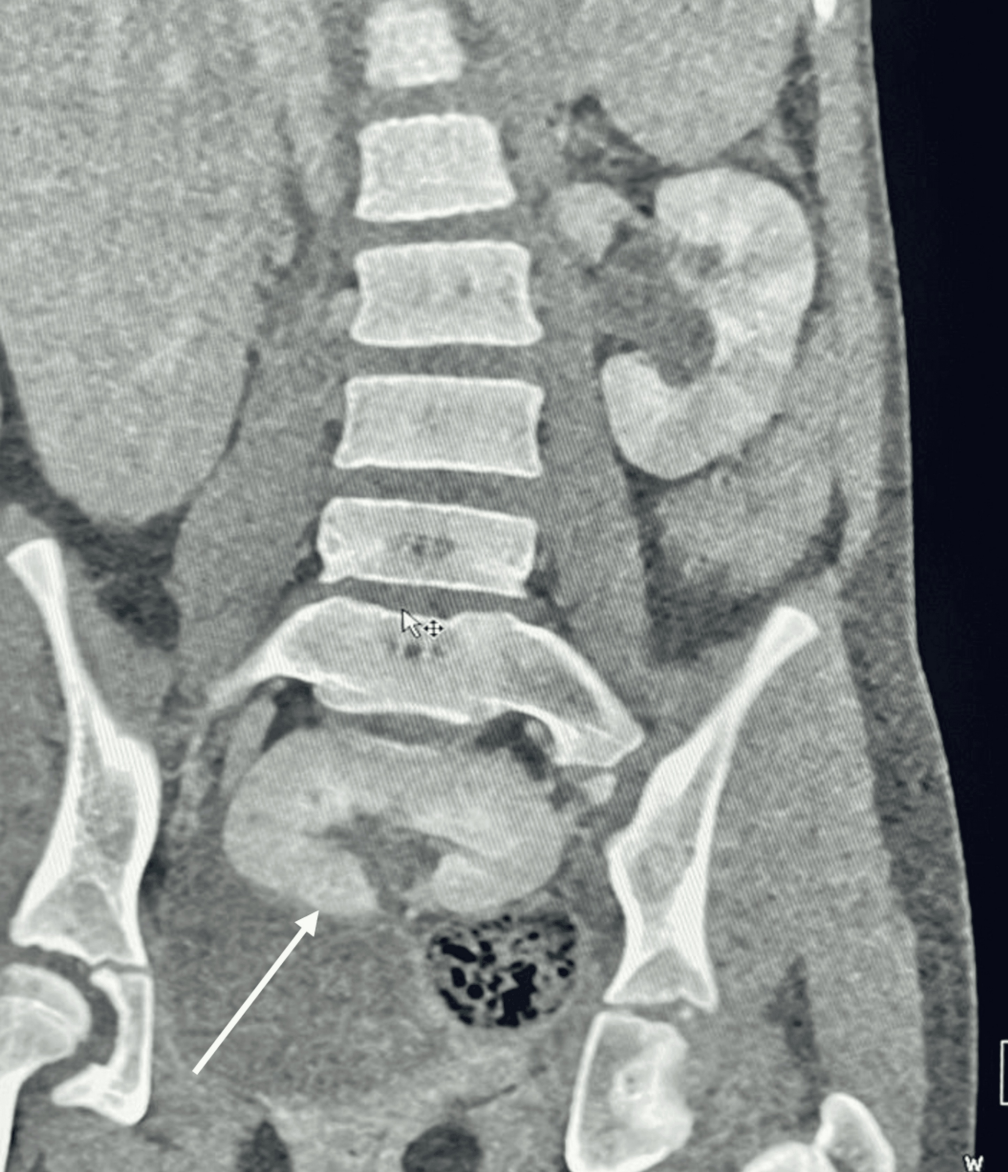
CECT abdomen showing ectopic right pelvic kidney The white arrow points to the ectopic right pelvic kidney. CECT, contrast-enhanced computed tomography.

The contrast-enhanced computed tomography (CECT) abdomen showed the right kidney located in the pelvis, anterior to the sacral curvature and posterior to the urinary bladder, with an anteriorly directed hilum. Normal functioning and excreting bilateral kidneys were found (Figure 2).

The renal dimercaptosuccinic acid (DMSA) scan showed mildly impaired cortical function in the mid-polar region, with a differential function of 35% of the left kidney. The right kidney is ectopic in location with preserved cortical function and no scars.

**Figure 3:**
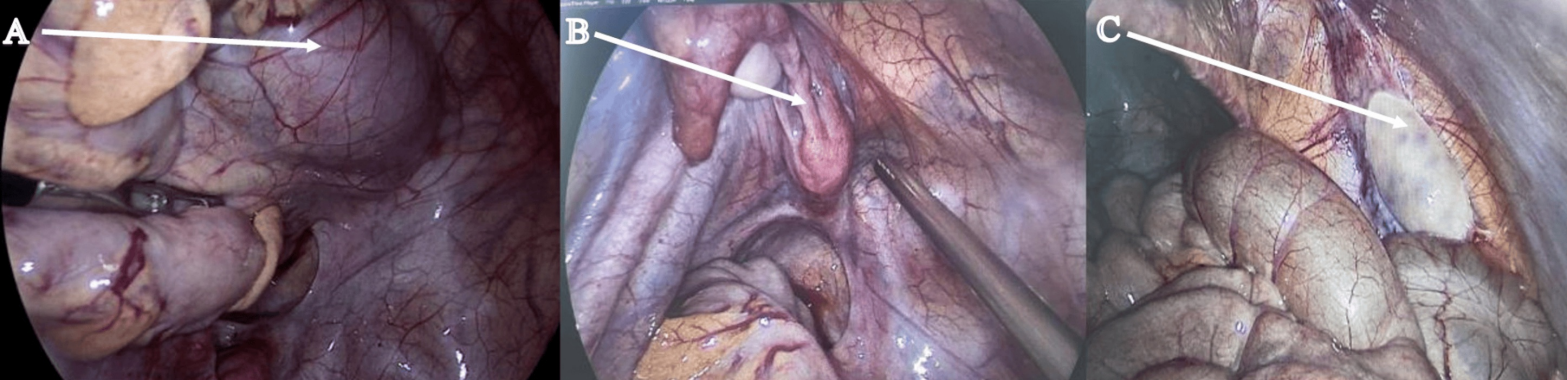
(A) Ectopic kidney. (B) Unicornuate uterus with left fallopian tube and ovary. (C) Right ectopic ovary located near the deep inguinal ring The white arrow in (A) points to ectopic right kidney located in the pelvis , while that in (B) points to unicornuate uterus with left fallopian tube and ovary and absent right fallopian tube and that in (C) points to ectopic right ovary near deep inguinal ring.

The patient was planned for laparoscopic left extravesical ureteric reimplantation. Under general anesthesia, cystoscopy was done. On cystoscopy, the right ureteric opening was central in location, and the left ureteric opening was found to be laterally placed. The left ureter was stented with a 4Fr, 26 cm DJ (Double-J) stent. Pneumoperitoneum was created with the Verres needle technique. A 10 mm umbilical port was inserted, and two 5 mm ports were inserted in the right and left lumbar regions. The patient was placed in the Trendelenburg position. On diagnostic laparoscopy, the right ectopic kidney was found in the midline with the hilum facing downward and the visible right ureter, a left-sided unicornuate uterus lying below the left iliac vessels, and an ectopic right ovary located near the right deep inguinal ring (Figure 3).

The left ovary and fallopian tube were seen in close relation to the uterus. There was no fallopian tube on the right side. The distal end of the left ureter was found in close relation to the uterus.

**Figure 4:**
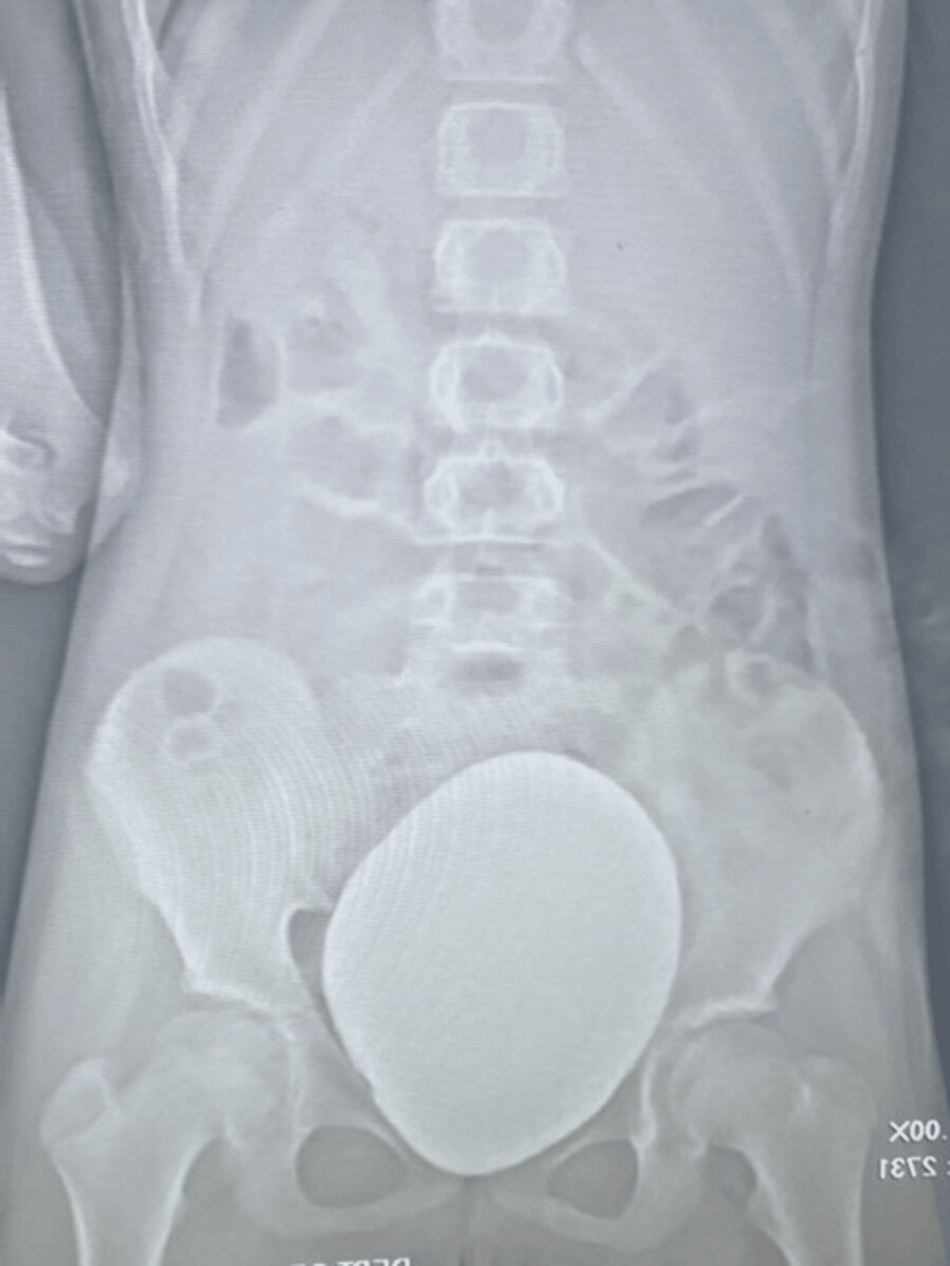
Postoperative MCUG after three months MCUG, micturating cystourethrogram.

The left ureter was identified in the distal part. The peritoneum over the ureter and bladder was divided. The ureter was dissected near the vesicoureteric junction and looped with a vessel loop (Figure 3C). Space was created in the broad ligament, which was closely attached to the uterus, and 3-4 cm of the ureteric length was mobilized. The bladder was partially filled with normal saline, and an incision was marked over the bladder with monopolar cautery. The detrusor muscle was divided and separated till the bladder mucosa popped out. The bladder was hitched to the anterior abdominal wall with two Ethilon 1-0. The mobilized ureter was placed in the detrusor tunnel, and detrusoraphy was done with Vicryl 4-0 in an interrupted fashion. Four interrupted sutures were applied to place the ureter in the detrusor tunnel. The postoperative period was uneventful, and the patient was discharged on postoperative day (POD) 4. The DJ stent was removed on POD 14. Repeat MCUG was done at three months, and there was no evidence of VUR (Figure 4).

## Discussion

Congenital renal anomalies and MDAs are often associated because of their common embryologic origin from the genital ridge, which develops concurrently during early gestation [2]. Disruptions occurring between the sixth and ninth weeks of gestation affect the development of both the urinary and reproductive systems. This shared embryologic association explained the rare combination of renal ectopia, VUR, unicornuate uterus, and ectopic ovary in our patient. 

Although these anomalies are well recognized, they are often overlooked in clinical practice, particularly in prepubertal girls who present primarily with urological complaints. In our patient, the presence of an ectopic kidney should have raised suspicion for an associated MDA; however, it was initially missed and was later identified during laparoscopy.

VUR has been reported in 30-50% of children with ectopic kidneys when MCUG is performed [5]. Reflux may occur in either the ectopic or the orthotopic kidney. Guarino et al. [5] reported that 86% of patients had reflux into the orthotopic kidney in cases of unilateral renal ectopia. Our patient had grade IV VUR in the orthotopic kidney with reduced function and renal scarring on DMSA. Cystoscopy showed a normally positioned ureteric orifice for the ectopic right kidney, while the left ureteric orifice was displaced laterally.

Before puberty, diagnosis of uterine and vaginal anomalies is challenging and may be misleading due to the small size of the prepubertal uterus and the absence of hormonal stimulation and menstrual vaginal distension [6]. During evaluation, a CECT abdomen was performed in our patient, but the findings of a unicornuate uterus and an ectopic ovary were missed. CECT has a very limited role in the evaluation of female genital tract congenital anomalies [7].

MRI is the standard imaging modality for comprehensive evaluation of Müllerian anomalies, as it provides superior soft-tissue resolution without radiation exposure [7]. MRI is a non-invasive imaging modality with a sensitivity of 100% to 28.6% and a specificity of 100% to 66% in correctly categorizing MDAs, making it very useful in pediatric and virgo intacta patients [7,8].

Diagnostic laparoscopy also plays an important role when surgical correction is planned, allowing direct visualization and simultaneous management of associated anomalies.

The right ovary of the patient was ectopically placed near the deep inguinal ring. Ombelet et al. [9] have shown the rare association of a unicornuate uterus and ectopic (undescended) ovary. The ovaries descend to their final position in the pelvis from their position near the kidneys, guided by the gubernaculum during the third month of fetal life. The gubernaculum attaches to the uterus, forming the round ligaments and utero-ovarian ligaments [8]. Due to uterine malformations, the gubernaculum may pull the ovary down in the canal of Nuck, potentially leading to inguinal or labial herniation in the absence of cornual attachment. Ovarian maldescent occurs in more than 40% of cases of a unicornuate uterus, which explains the ectopic location of the right ovary in our patient [8].

The initial presentation in patients with isolated MDAs commonly occurs at puberty with dysmenorrhea or primary amenorrhea. Our patient had a type 2 Müllerian anomaly, which is due to incomplete development of one Müllerian duct (unicornuate uterus). The incidence of this anomaly is about 9-10% of all Müllerian anomalies and is associated with ipsilateral renal abnormalities [10]. The patient with a unicornuate uterus has an increased risk of obstetric complications such as ectopic pregnancy, early miscarriages, intrauterine growth retardation, abnormal fetal presentation, and preterm labor [10].

In prepubertal girls with multiorgan anomalies, the initial presentation is often urologic, commonly manifesting as urinary incontinence or retention, pelvic pain, pelvic mass, or infection [1]. The presentation in our case was pain in the lumbar region and urinary infection. In our case, the patient has not achieved a menarche and is in Tanner stage 1. In our patient, laparoscopic extravesical reimplantation was successfully performed, with complete resolution of VUR on follow-up MCUG (Figure 4).

## Conclusions

This case highlights the intricate embryological relationship between the genitourinary systems. Awareness of the association between Müllerian and renal anomalies in female patients is essential for optimal preoperative imaging, surgical planning, and counseling regarding future gynecological and obstetric implications.
